# Predictors and associations of complications in ureteroscopy for stone disease using AI: outcomes from the FLEXOR registry

**DOI:** 10.1007/s00240-025-01763-8

**Published:** 2025-05-14

**Authors:** Carlotta Nedbal, Vineet Gauhar, Sairam Adithya, Pietro Tramanzoli, Nithesh Naik, Shilpa Gite, Het Sevalia, Daniele Castellani, Frédéric Panthier, Jeremy Y. C. Teoh, Ben H. Chew, Khi Yung Fong, Mohammed Boulmani, Nariman Gadzhiev, Thomas R. W. Herrmann, Olivier Traxer, Bhaskar K. Somani

**Affiliations:** 1 Polytechnic University Le Marche, Ancona, Italy; 2 Ng Teng Fong General Hospital, Urology, Singapore, Singapore; 3 Symbiosis Institute of Technology, Engineering, Pune, India; 4 Manipal Academy of Higher Education, Engineering, Manipal, India; 5 Azienda Ospedaliero-Universitaria Ospedali Riuniti Di Ancona, Polytechnic University Le Marche, Ancona, Italy; 6 GRC Urolithiasis No. 20, Sorbonne University, Tenon Hospital, Paris, France; 7 Urology, The Chinese University of Hong Kong, Hong Kong, China; 8 Urology, University of British Columbia, Vancouver, Canada; 9 Urology, Yong Loo Lin School of Medicine, National University of Singapore, Singapore, Singapore; 10 Boston Scientific – Urology and Pelvic Health, Paris, France; 11 Pavlov First Saint Petersburg State Medical University, Saint Petersburg, Russia; 12 Kantonspital Frauenfeld, Spital Thurgau AG, Frauenfeld, Switzerland; 13 Sorbonne University GRC Urolithiasis No. 20, Tenon Hospital, Paris, France; 14 University Hospital Southampton NHS Foundation Trust, Southampton, UK; 15 Urology, ASST Fatebenefratelli Sacco, Milan, Italy; 16 Endourology Section, European Association of Urology, Arnhem, The Netherlands; 17 IRCSS San Gerardo, Monza, Italy; 18 Progressive Endourological Association for Research and Leading Solutions (PEARLS), Paris, France; 19 UMR 8006 CNRS-Arts Et Métiers ParisTech, PIMM, Paris, France

**Keywords:** Urolithiasis, Machine learning, Outcomes, Predictive model, Explainable AI

## Abstract

We aimed to develop machine learning(ML) algorithms to evaluate complications of flexible ureteroscopy and laser lithotripsy(fURSL), providing a valid predictive model. 15 ML algorithms were trained on a large number fURSL data from > 6500 patients from the international FLEXOR database. fURSL complications included pelvicalyceal system(PCS) bleeding, ureteric/PCS injury, fever and sepsis. Pre-treatment characteristics served as input for ML training and testing. Correlation and logistic regression analysis were carried out by a multi-task neural network, while explainable AI was used for the predictive model. ML algorithms performed excellently. For intraoperative PCS bleeding, Extra Tree Classifier achieved the best accuracy at 95.03% (precision 80.99%), and greatest correlation with stone diameter(0.21) and residual fragments(0.26). PCS injury was best predicted by RandomForest (accuracy 97.72%, precision 63.50%). XGBoost performed best for ureteric injury (accuracy 96.88%, precision 60.67%). Both demonstrated moderate correlation with preoperative characteristics. Postoperative fever was predicted by Extra Tree Classifier with 91.34% accuracy (precision 58.20%). Cat Boost Classifier predicted postoperative sepsis with 99.15% accuracy (precision 66.38%), and the best overall performance. At logistic regression, postoperative fever/sepsis positively correlated with preoperative urine culture(p = 0.001). ML represents a powerful tool for automatic prediction of outcomes. Our study showed promises in algorithms training and validation on a very large database of patients treated for urolithiasis, with excellent accuracy for prediction of complications. With further research, reliable predictive nomograms could be created based on ML analysis, to serve as aid to urologists and patients in the decision making and treatment planning process.

**Supplementary Information:**

The online version contains supplementary material available at 10.1007/s00240-025-01763-8.

## Introduction

A recent review of epidemiological data from seven countries revealed incidence rates for kidney stones of 114–720 per 100,000 individuals and prevalence rates of 1.7–14.8%, and is increasing worldwide, mainly due to the association with metabolic disorders and ageing of population [1]. During the last decades, the surgical management of kidney stones benefited of many technological advances and one of them is the development of flexible ureteroscopy (fURS) [2–4]. fURS with laser lithotripsy represent the gold standard for stones smaller than 2 cm [5]. Although managing stone disease with endoscopic procedures is a common practice for urologists, these surgeries carry a considerable risk of complications. In some cases, these complications can be clinically significant, with potential critical implications for patients in the long-term follow-up [6].

The most frequent complications following ureteroscopy in the peri- and post-operative period are fever (2–28%) and sepsis (3–5%), steinstrasse (1%), and ureteral injury [7–9], but these complications are usually classified as Clavien-Dindo I and II or sometimes IIIa, when an endoscopic intervention is required without general anesthesia. Rare complications include ureteral avulsion (< 1%), ureteral strictures (2–3%), kidney damage, fistulas and severe bleeding with transfusion [10, 11]. Several factors seem to increase the likelihood of post-operative fever and sepsis. These include having a positive pre-operative urinary tract infection (UTI) or a history of recurrent UTIs, a higher Charlson comorbidity index, elderly patients or female gender. Other risk factors include the presence and duration of ureteral stents, longer procedural times, neurogenic bladder conditions, and a high body mass index (BMI) [6, 12, 13].

In the prevention of complications related to fURS, it may be beneficial to take advantage of technological evolution in the field of artificial intelligence [14]. Artificial intelligence (AI) is revolutionizing the field of medicine, that is in enhancing diagnostics, treatment planning, and patient care. AI-driven algorithms, particularly in machine learning (ML) and deep learning, are now used to analyze medical images, identify patterns in complex datasets, and provide insights that aid in early diagnosis, such as detecting cancer in radiology scans or identifying anomalies in pathology slides [15]. A particularly noteworthy application is leveraging ML as a predictive tool for postoperative outcomes [16]. The capacity to anticipate postoperative complications, predict recovery paths, and assess overall patient well-being signifies a transformative change in surgical healthcare [17].

The aim of this study is to develop ML algorithms for the automated prediction and association of complications following ureteroscopy for kidney stones, based on preoperative characteristics.

## Materials and methods

1. FLEXOR registry: Patients selection, data collection and procedures.

FLEXOR, an international multicentric database, collected practice patterns and outcomes of 6669 patients treated with fURS for urolithiasis from 2015 to 2023, and data was subsequently analysed. Twenty centers from fifteen countries enrolled adult patients (> 18 years old) who underwent fURS for urolithiasis. Preoperative and demographic characteristics were collected, including age, gender, presentation (acute, at follow-up, incidental), symptoms, comorbidities, stone size, total stone burden (cumulative length), stone number and locations, urine culture results, presence of a preoperative urinary drainage. Additionally, the presence of preoperative haematuria, fever, positive urine culture and the use of alpha-blocker medications (Tamsulosin) was recorded. Preoperative imaging consisted of plain or contrast-enhanced CT scan to evaluate anatomy and stone features. Patients undergoing fURS for other diagnosis than urolithiasis (i.e., upper tract tumour), paediatric patients, and patients not giving consent for data collection were excluded from the study. Every patient received specialistic counselling before surgery, an informed consent and permission towards data registration were routinely obtained. Antibiotic prophylaxis was carried out according to local protocols and was not standardised among the centers.

During surgery, information was recorded on: type of anaesthesia (spinal, epidural, general), operative time (from instrument insertion to catheterisation), instrumentations (ureteric access sheath (UAS), single-use/reusable scope, digital/fibreoptic scope, use of baskets), source of energy and mode (laser type, MOSES, dusting/fragmentation), lasering time, need for postoperative stent insertion. Intraoperative complications such as pelvicalyceal system (PCS) or ureteric injury, PCS bleeding and need for intraoperative transfusion were also recorded. Early postoperative complications including fever, sepsis, haematuria, pain, were monitored and classified according to the postoperative Clavien-Dindo scale. For each patient, hospitalisation days and readmissions were noted. At follow-up, stone free status (SFS) was assessed according to the local protocol (KUB X-Ray and/or ultrasound or non-contrast CT scan). SFS was defined as absence of fragments > 2 mm in the urinary tract.

2. ML algorithms training and data analysis.

Work flow involved several stages, starting with data cleaning and preprocessing. Initially, the data were cleaned by removing unnecessary spaces and irrelevant characters. Following this, preprocessing steps were applied, such as imputing mode values for categorical preoperative characteristics. Next, we performed statistical analyses, which included evaluating correlations, assessing variance inflation factors (ViF), and conducting logistic regression for all four tasks. Afterwards, we trained individual ML algorithms for each task, while simultaneously developing a multitask artificial neural network (ANN) to handle all tasks together. We then applied explainable AI techniques to provide insights into the predictions generated by the models.

A total of sixteen different ML algorithms were selected and trained on the processed data for each of the four tasks. These included logistic regression, quadratic discriminant analysis, Extra Trees Classifier, Adaboost, Cat Boost Classifier, Naïve Bayes, Bagging Classifier, Gradient Boost, Extreme Gradient Boost (XGBoost), Decision Tree, K-Nearest Neighbour (KNN), Random Forest, Linear Discriminant Analysis, and Support Vector Machine (SVM) with linear, polynomial, and radial basis function kernels.

Each of these algorithms was trained separately to predict outcomes based on input features for the tasks.

- Inputs: Age; Sex; Preoperative (PreOp) Haematuria; PreOp Pain; PreOp Elevated creatinine; PreOp Fever; PreOp Positive urine culture; PreOp Stent; Use of Tamsulosin; Normal/abnormal anatomy of urinary tract; Single/multiple stone; Stone diameter; Location of stone; Use of UAS; Suction UAS; Reusable/disposable scope; Digital/fibre optic scope; Use of pulse modulation with Moses technology; Use of thulium fiber laser (TFL); Insertion of stent/percutaneous nephrostomy (PCN); Residual fragment(s).
- Outputs: intraoperative complications (PCS bleeding; PCS injury; Ureteric injury), infectious complications (Postoperative fever; Sepsis).

For multitasking, we used algorithms capable of making multiple predictions at once. Specifically, a multitask ANN was built to predict all postoperative outcomes simultaneously. This network featured shared layers for initial feature extraction, followed by task-specific layers. The architecture included an input layer with eight neurons, a shared hidden layer of 128 neurons with a rectified linear unit (ReLU) activation function, and four separate output layers with one neuron each, employing a sigmoid activation function for predicting each postoperative outcome.

The performance of the ML models was primarily evaluated using a confusion matrix and a classification report. The confusion matrix provided values for true positives, true negatives, false positives, and false negatives, which were used to calculate metrics such as accuracy, precision, recall, and the F1 score. The classification report offered precision and recall metrics for each class within the task.

In recent years, the importance of interpretability and explainability in predictive models has grown, especially in sensitive fields like healthcare. Complex algorithms like ANNs, often considered"black boxes,"require methods that enhance transparency. Various techniques, such as tree explainer, feature importance, and Shapley additive explanations (SHAP) were utilized to provide clear interpretations of the model predictions by highlighting key features and their contributions to the outcomes.

## Results

### Results from the FLEXOR registry

The results from FLEXOR registry have already been published on behalf of the TOWER research group [18]. We therefore provide only a brief description of these (a complete overview of the results can be found in the supplementary materials). A total of 6669 patients have been enrolled in the study, involving 20 centers internationally. Women accounted for 33.8% and men for 66.2% of cases. Mean patients age was 49.3 ± 15.59 years. Most of the patients presented with pain (62.6%), while a small cohort was diagnosed incidentally (10.2%). The majority of cases had a single stone (59.0%), and the mean stone diameter was 10.04 ± 6.84 mm. fURSL was preferably performed with a reusable flexible ureteroscope in 4803 (72.0%) procedures, and a UAS was used in 93.2% of the cases, leading to intraoperative ureteral injury in 1.8% of the patients. The Holmium:YAG laser was used in 4878 cases (73.1%), with a combination of dusting and fragmentation being the most frequently used lithotripsy technique (64.3%). The average operation time was 62.40 ± 17.76 min, and mean hospitalisation length was 3.62 ± 3.47 days. Postoperative complications occurred in 535 patients (8.0%), with 84 patients (1.3%) developing sepsis requiring intensive care admission. At follow-up, 78.3% of the patients were stone free. Among the 1445 (21.7%) with residual fragments, 744 (51.5%) patients required additional intervention.

### Prediction of intraoperative complications

Intraoperative complications, namely significant bleeding from PCS, ureteric or PCS injury, were analysed separately through correlation and logistic analysis. Complete AI findings are shown in the supplementary materials, with detailed description of ML results (including precision and recall), correlation analysis and statistical analysis, alongside explainable AI figures.

A. PCS bleeding

Among the ML algorithms, the Extra Tree Classifier was the best performer in both training and validation set, reaching a validation accuracy of 95.03% and a precision of 80.99%. XG Boost (accuracy 94.60%, precision 79.24%) and Random Forest (accuracy 94.46%, precision 79.17%) were respectively the second and third performing algorithms. The correlation between PCS bleeding and the other features is shown by the explainable AI: according to the Explainable Tree, the features used for prediction are: use fibreoptic or reusable scope, stone diameter, UAS, stone located in the middle pole and presence of multiple stones, with fibreoptic scope having the highest priority. This indicates that these features are highly important and contribute to the diagnosis of PCS bleed.

B. PCS injury

Random Forest was the best performing algorithm in the prediction of PCS injury in both training and testing, with an accuracy of 97.73% and a precision of 63.50%. Extra Tree Classifier followed with an accuracy of 97.02% and a precision of 54.14%. Explainable AI shows the correlation between PCS injury and other inputs: as depicted by the Explainable Tree, the use of a digital scope, of TFL, of larger UAS and the presence of higher stone burden are considered highly important and contribute to the prediction of PCS injury.

C. Ureteric injury

ML algorithms testing and training revealed the XG Boost as the best performing algorithm, with a validation accuracy of 96.88% and a precision of 59.27%. The second and third best algorithms were Cat Boost Classifier (accuracy 96.31%, precision 57.95%) and Random Forest (accuracy 96.16%, precision 57.46%) respectively. Analysing the Explainable Tree in the Explainable AI results, ureteric injury was highly correlated with presence of preoperative pain, stone located in the middle/upper pole or renal pelvis, and the use of larger UAS or reusable scopes. This indicates that these features are highly important and contribute to the prediction of ureteric injury.

### Infectious complications

A. Postoperative fever

On correlation analysis,'Postoperative fever'showed a weak negative correlation with'pain at presentation'(−0.010),'normal anatomy'(−0.032),'multiple stones'(−0.002),'UAS'(−0.029),'suction UAS'(−0.015),'reusable scope'(−0.028), and'TFL'(−0.024). A positive correlation was demonstrated with'age'(0.035),'male sex'(0.053),'elevated creatinine','fever'(0.101),'positive preoperative urine culture'(0.157),'preoperative stent'(0.053),'tamsulosin'(0.059),'moses fibre'(0.014),'increasing stone diameter'(0.067), and stone location:'upper pole'(0.019),'middle pole'(0.034),'lower pole'(0.007), and'renal pelvis'(0.005). These results suggest that preoperative urine culture plays the main role in the occurrence of postoperative fever, while factors such as age, sex, preoperative elevated creatinine, fever, urine culture, preoperative stent, use of tamsulosin, stone characteristics (diameter, location), and the use of moses fibre may be associated with its incidence.

Logistic regression results for'postoperative fever'indicated that the variables'elevated creatinine'(0.719, p = 0.001),'fever'(0.626, p = 0.004),'positive preoperative urine culture'(1.305, p < 0.001),'tamsulosin'(0.445, p = 0.042),'multiple stones'(−0.477, p = 0.020),'increasing stone diameter'(0.047, p < 0.001), and stone located in the'middle pole'(0.434, p = 0.021) have positive coefficients, being associated with the likelihood of this complication. This suggests that higher values of these variables increase the odds of'postoperative fever'. Conversely, variables like'reusable scope'(−0.522, p = 0.009) and'TFL'(−0.448, p = 0.035) have negative coefficients, indicating that higher values of these variables decrease the odds of'postoperative fever'. Some variables, such as'age','sex','preoperative haematuria','pain at presentation','preoperative stent','normal anatomy','UAS','suction UAS','fibreoptic scope', and'Moses Fibre', do not appear to have a significant association with'postoperative fever'.

**Fig. 1:**
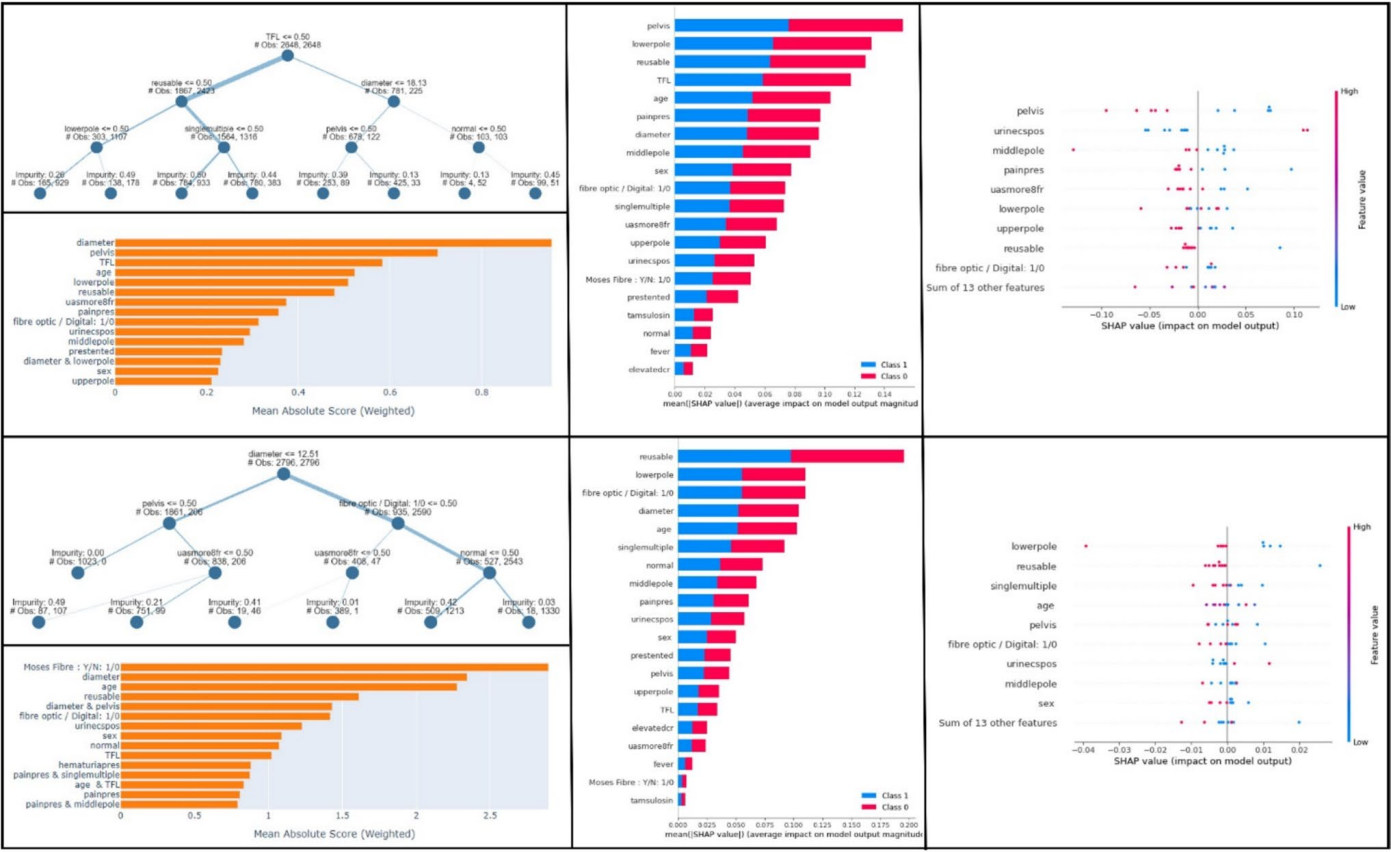
Explainable AI. In the upper half: explainable AI for prediction of postoperative fever (from the upper left: Explainable Tree, Feature Importance Plot, Shap Bar Chart, Chap Beeswarm Chart). In the lower half: explainable AI for prediction of postoperative sepsis (from the upper left: Explainable Tree, Feature Importance Plot, Shap Bar Chart, Chap Beeswarm Chart)

Among the 15 ML algorithms, the best performing in prediction of postoperative fever was the Extra Tree Classifier, with a validation accuracy of 91.34% and a precision of 58.20%. XG Boost and Random Fores followed with slightly inferior values: accuracy 91.34% and precision 54.07% for the XG Boost, accuracy 91.05% and precision 57.67% for the Random Forest. Explainable AI (Fig. 1) was used to show correlation between postoperative fever and other features. The Explainable Tree shows how the use of TFL, reusable scopes, the presence of larger, multiple stones or stones located in the lower pole or the renal pelvis had the highest priority and represented the most important features for prediction of postoperative fever.

B. Postoperative sepsis

Cat Boost Classifier was the best performing ML algorithm in the prediction of postoperative sepsis, with a high validation accuracy of 99.01% and validation precision of 66.45%. Random Forest and Extra Tree Classifier reached the same validation accuracy (99.15%) and precision (66.38%), representing the following best models. Explainable AI (Fig. 1) shows correlation features for the incidence of postoperative sepsis. According to the Explainable Tree, the features used for prediction are stone diameter, stone located in the renal pelvis, the use of fibreoptic scope and the lack of UAS, with stone diameter having the highest priority. It indicates that these features are highly important and contribute to the prediction of postoperative sepsis.

## Discussion

This study is the first ML-driven evaluation of complications and their correlations in a large cohort of patients undergoing fURSL for urolithiasis. We explored 15 different ML algorithms, training and testing them on a very large data set, to accurately predict complications based on preoperative and intraoperative features, with a focus on infectious complications. Models like the Extra Tree Classifier, Random Forest, and Cat Boost Classifier achieved high accuracy in predicting complications, especially PCS bleeding and postoperative sepsis, suggesting potential interesting application of ML in urology. In particular, Cat Boost Classifier showed the highest overall performance, with an accuracy of 99% for postoperative sepsis, which brings great promise for the accurate prediction of infectious complication following stone treatments.

Our analysis showed significant correlation between preoperative urine culture and postoperative fever and sepsis (p = 0.001). This finding, far from being revolutionary as the importance of performing fURS in a sterile urine tract has already been investigated [19], underscores the clinical relevance of infection control and patient preparation, particularly in individuals identified as higher-risk through preoperative urine culture results. In fact, despite having received a preoperative course of tailored antibiotic therapy, patient with an initial positive urine culture still showed increased odds of developing postoperative infectious complications.

According to this ML-driven analysis, other factors such as overall stone burden and presence of residual fragments were noted to have lower correlation values with the considered outcomes. Despite being modest and not showing strong inference on complications, these characteristics could still play a role in their development, though perhaps as part of a broader set of predictive features. Considering this, even moderate correlation features could be considered for the development of a punctual nomogram for ureteroscopy complications and outcomes prediction.

To emphasise our findings, XAI results have been proposed. XAI is the field within computer science focused on making"black-box"models transparent, traceable, and understandable. This area is gaining significance, especially as quality standards for medical AI solutions are rising. Human experts now need to not only interpret machine-generated decisions but also understand the reasoning behind them, rather than relying solely on conventional metrics like accuracy and precision [20]. These AI tools can increase trust in the models and enable clinicians to make more informed, data-driven decision. Being visually informative and catchy, XAI models can also help during patient consultations, increasing risks communications and tailored treatment discussion, serving as a reference for the individual risk profile [21]. In our analysis, several XAI models such as explainable tree and SHAP models have been tested with good results; i.e., the Explainable Tree for prediction of ureteric injury shows correlation with preoperative and intraoperative characteristics such as specific stones locations and the use of larger UAS. While some XAI models still pose challenges for clinicians not familiar with AI, we believe that XAI will gain more and more appeal for urologists, in both research and clinical practice, providing a comprehensive and informative picture of outcomes correlations for different procedures and diagnosis.

Application of ML in urology is quite a novelty in research, with few studies reporting on similar results. Pietropaolo and colleagues discussed the application of ML in predicting sever sepsis post fURS in a smaller cohort of 114 patients [22]. They found association with proximal stone location, long stent time, large stone size and long operative time, obtaining good accuracy profiles at 81.3%. Recently, another study on over 1500 patients was conducted by Castellani et al. to investigate possible predictors of post-ureteroscopy sepsis [23]. Similarly, they found associations with patient’s age, stone volume and operative time, with good accuracy at 92%. Other studies investigated surgical outcomes of ureteroscopy by using ML algorithms, though not focusing on complications. In these investigations, ML analysis underscored correlation between stone free status and total stone burden [24, 25], or was applied to predict stone composition based on intraoperative features [26, 27].

One of the main strengths of our study is indeed the very large number of data analyses, from the FLEXOR database including over 6500 patients from different centers. While variability in surgical technique, instrumentation, and perioperative protocols may vary among the centers, impacting outcomes, the authors believe that the vastity of characteristics and information obtained from this database make our analysis reliable and reproductive of real-world situation. Moreover, we trained and tested 15 different ML algorithms, thus gaining better combinations and obtaining excellent results from the best performing ones. Nevertheless, our study is not without limitation. First of all, the retrospective nature of the database, with consequent risk of missing or incomplete data. Moreover, the multicentricity of our resources might bring some biases from different procedural steps and instrumentations, but at the same time we believe it to be reflective of the worldwide practice. Lastly, we have to acknowledge that our analysis, despite achieving great overall accuracy for the set outcomes, showed only moderate precision in the prediction of some complications, namely postoperative fever (58.20%) and ureteric injury (60.67%). This suggests that while these models can effectively flag risks, their reliability in clinical settings could still be improved. Further studies with strategies to enhance precision, such as targeted feature engineering and model calibration, would improve precision values, and their implications for clinical decision-making.

Finally, there is a need to highlight some ethical issues that come from the implementation of AI in healthcare raises, including data privacy and the need for rigorous validation before deployment in clinical settings [28, 29]. In our work, all data were rigorously anonymised and respected patient privacy, but with shared ML algorithms and spread of AI models, there will be a need for standardised privacy and transparency standards and regulatory oversight.

We believe that future work could focus on refining ML models for higher precision to ensure robust validation, and research could explore integrating real-time intraoperative data to further enhance prediction accuracy for complications as the procedure unfolds. Moreover, long-term studies could assess how predictive models affect patient outcomes, cost-efficiency, and overall patient satisfaction in the clinical decision-making process.

## Conclusion

ML represents a powerful tool for automatic prediction of outcomes. Our study showed promises in algorithms training and validation on a very large database of patients treated for urolithiasis, with excellent accuracy for prediction of complications. With further research, reliable predictive nomograms could be created based on ML analysis, to serve as aid to urologists and patients in the decision making and treatment planning process.

## Supplementary Information

Below is the link to the electronic supplementary material.

Supplementary file1 (DOCX 747 KB)

## Data Availability

Complete database and analysis are available upon request to the authors.
