# Supplementary material for: Predictors and associations of complications in ureteroscopy for stone disease using AI: outcomes from the FLEXOR registry
Source: Urolithiasis. 2025 May 14;53(1):89. doi: 10.1007/s00240-025-01763-8 (PMC12078356; doi:10.1007/s00240-025-01763-8)
Supplement: Supplementary file 1 — Supplementary file1 (DOCX 747 KB) [file 240_2025_1763_MOESM1_ESM.docx]

**SUPPLEMENTARY MATERIALS**

1. **RESULTS FROM FLEXOR REGISTRY**

| **Characteristics** | **Numbers** |
| --- | --- |
| **Number of patients** | 6669 (100%) |
| **Age, years, mean (standard deviation)** | 49.34 (15.59) |
| < 40 years old | 2111 (31.6%) |
| 41–65 years old | 3494 (52.4%) |
| 66–75 years old | 731 (11.0%) |
| > 75 years old | 331 (5.0%) |
| **Gender (%)** |  |
| Male | 4407 (66.1%) |
| Female | 2262 (33.9%) |
| **Ethnicity** |  |
| Asian | 4225 (63.4%) |
| Non-Asian | 2444 (36.7%) |
| **First presentation of stone** | 5036 (75.5%) |
| **Symptoms on presentation** |  |
| Haematuria | 327 (4.9%) |
| Pain | 4178 (62.6%) |
| Elevated creatinine | 612 (9.2%) |
| Fever | 651 (9.8%) |
| Incidental Finding of Stone | 679 (10.2%) |
| Pre-stented | 3112 (46.7%) |
| Preoperative Tamsulosin Used | 1142 (17.1%) |
| **Diagnostic imaging modality** |  |
| CT Scan | 5094 (76.4%) |
| Contrast-enhanced CT scan | 814 (12.2%) |
| X-ray | 1562 (23.4%) |
| Ultrasound | 3294 (49.4%) |
| **Stone characteristics°** |  |
| HU, mean (standard deviation) | 978.94 (333.1%) |
| Multiple stones | 2732 (41.0%) |
| Size, mm, mean (standard deviation) | 10.04 (6.84) |
| Upper pole | 1474 (22.1%) |
| Mid pole | 2041 (30.6%) |
| Lower pole | 2946 (44.2%) |
| Renal pelvis | 2196 (32.9%) |
| **CT**: computed tomography, **HU**: Hounsfield Unit. | |

Table 1. FLEXOR Registry. Patients’ baseline characteristics

| **Perioperative parameters** | **Number** |
| --- | --- |
| **Preoperative antibiotics** | 5129 (76.9%) |
| **Urethral access sheath size** |  |
| 8 Fr | 1223 (18.3%) |
| > 8 Fr | 4992 (74.9%) |
| No use of sheath | 454 (6.8%) |
| **RIRS scope type** |  |
| Reusable | 4808 (72%) |
| Disposable | 1855 (27.8%) |
| **Use of Holmium Laser** | 4878 (73.1%) |
| **Use of Moses technology** | 516 (19.1%) |
| **Thulium fiber laser** | 1791 (26.9%) |
| **Lithotripsy technique** |  |
| Dusting | 3960 (59.4%) |
| Popcorning | 2337 (35%) |
| Fragmentation | 2611 (39.2%) |
| Combination | 4287 (64.3%) |
| **Laser Time, minutes, mean (standard deviation)** | 25.90 (17.76) |
| **Operation Time, minutes, mean (standard deviation)** | 62.40 (38.77) |
| **Intraoperative Complications** |  |
| PCS bleeding not requiring blood transfusion | 300 (4.5%) |
| PCS bleeding requiring blood transfusion (Clavien grade 2) | 6 (0.1%) |
| Ureteric injury due to access sheath requiring stenting (Clavien grade 3) | 119 (1.8%) |
| **URS** ureteroscopy, **Fr** French, **RIRS** retrograde intrarenal surgery, **W** Watt, **PCS** pelvicalyceal system. | |

Table 2. FLEXOR Registry. Intraoperative outcomes of 6669 cases enrolled in global FLEXOR study.

| **Characteristics** | **Numbers** |
| --- | --- |
| **Postoperative stay, days, mean (standard deviation)** | 3.62 (3.47) |
| **Day surgery** | 754 (11.3%) |
| **Overall postoperative complications** | 535 (8.0%) |
| Fever/Infections requiring antibiotics (Clavien grade 2) | 407 (6.1%) |
| Haematuria requiring blood transfusions (Clavien grade 2) | 366 (5.5%) |
| Sepsis requiring ICU admission (Clavien Grade 4) | 84 (1.3%) |
| **Post-operative imaging assessment by** |  |
| CT scan | 1748 (26.2%) |
| X-ray | 2980 (44.7%) |
| Ultrasound | 3024 (45.3%) |
| Combination | 1942 (29.1%) |
| **Residual fragments** | 1445 (21.7%) |
| **Residual fragment subsequent treatment (n = 1445)** |  |
| SWL | 257 (17.8%) |
| RIRS | 400 (27.7%) |
| PCNL | 65 (4.5%) |
| ECIRS | 22 (1.5%) |
| Observation alone | 701 (48.5%) |
| **Stone analysis** | 2845 (42.7%) |
| **ICU** intensive care unit, **CT** computed tomography, **SWL** Shock Wave Lithotripsy, **RIRS** Retrograde Intrarenal Surgery, **PCNL** percutaneous nephrolithotomy, **ECIRS** Endoscopic Combined Intrarenal Surgery | |

Table 3. FLEXOR Registry. Postoperative outcomes of 6669 cases enrolled in global FLEXOR study

1. **STATISTICAL ANALYSIS – CORRELATION ANALYSIS**

| **Variable** | **Correlation with PCS bleed** | **Correlation with PCS injury** | **Correlation with Postoperative Drainage** | **Correlation with Ureteric Injury** |
| --- | --- | --- | --- | --- |
| Age | -0.003170 | -0.004863 | 0.138228 | -0.014135 |
| Sex | -0.040892 | 0.011463 | 0.036181 | 0.004986 |
| Haematuria | -0.044279 | -0.022638 | 0.080924 | -0.010728 |
| Pain at presentation | -0.066932 | -0.017943 | -0.148337 | -0.071161 |
| Elevated S-creatinine | 0.246391 | 0.068325 | 0.176771 | 0.114368 |
| Fever | 0.107065 | 0.103861 | 0.088773 | 0.098726 |
| Positive urine culture | -0.075773 | -0.034164 | 0.092885 | -0.008808 |
| Prestented | 0.061773 | -0.029140 | 0.124605 | 0.033496 |
| Use of tamsulosin | -0.084951 | -0.041432 | 0.231407 | 0.014848 |
| Normal anatomy | 0.046825 | 0.015679 | -0.108228 | 0.011009 |
| Number of stone | 0.055558 | 0.007743 | 0.246163 | 0.072390 |
| Stone diameter | 0.205208 | 0.023892 | -0.092386 | -0.020274 |
| Upper pole stone | -0.004478 | -0.020369 | 0.133001 | 0.010968 |
| Middle pole stone | -0.104171 | -0.059587 | 0.032831 | -0.012267 |
| Lower pole stone | 0.096668 | 0.083940 | 0.250458 | 0.091035 |
| Pelvic stone | 0.113092 | 0.006671 | -0.101690 | 0.003689 |
| UAS size | 0.042648 | -0.004020 | -0.151269 | -0.034633 |
| Suction UAS | -0.019192 | -0.012045 | -0.069106 | -0.016245 |
| Reusable scope | -0.069234 | -0.006156 | 0.165452 | -0.003275 |
| Fibreoptic scope | -0.190538 | -0.094738 | -0.129643 | -0.046743 |
| Moses Fibre | -0.087458 | -0.034600 | 0.254627 | 0.029864 |
| TFL | -0.091666 | -0.054918 | -0.290501 | -0.042145 |
| PCS bleed | 1.000000 | 0.250529 | 0.197139 | 0.146717 |
| PCS injury | 0.250529 | 1.000000 | 0.074861 | 0.168057 |
| Postoperative drainage | 0.197139 | 0.074861 | 1.000000 | 0.172981 |
| Ureteric injury | 0.146717 | 0.168057 | 0.172981 | 1.000000 |
| Postoperative fever | 0.060196 | 0.003802 | 0.079495 | 0.026067 |
| Postoperative sepsis | 0.067505 | 0.050603 | 0.011296 | 0.032145 |
| Residual fragments | 0.255168 | 0.097821 | 0.247951 | 0.090794 |
| Reintervention | 0.327595 | 0.123861 | 0.243619 | 0.082168 |
| Day surgery | -0.071500 | -0.007552 | -0.135674 | -0.012063 |

Table 4. Correlation analysis with intraoperative complications: PCS bleeding, PCS injury, insertion of Postoperative Drainage, Ureteric Injury

| **Variable** | **Correlation with Postoperative Fever** | **Correlation with Postoperative Sepsis** |
| --- | --- | --- |
| Age | 0.035456 | 0.031412 |
| Sex | 0.052906 | 0.019708 |
| Haematuria | 0.000757 | -0.016825 |
| Pain at presentation | -0.010496 | -0.023600 |
| Elevated S-creatinine | 0.065936 | -0.003680 |
| Fever | 0.100775 | 0.036178 |
| Positive urine culture | 0.156843 | 0.073836 |
| Prestented | 0.052945 | 0.019713 |
| Use of tamsulosin | 0.052945 | 0.063918 |
| Normal anatomy | -0.032321 | -0.055386 |
| Number of stone | -0.001515 | -0.005625 |
| Stone diameter | 0.066745 | 0.102052 |
| Upper pole stone | 0.019191 | 0.015551 |
| Middle pole stone | 0.034342 | 0.002102 |
| Lower pole stone | 0.007514 | -0.005865 |
| Pelvic stone | 0.005471 | 0.047086 |
| UAS size | -0.028827 | 0.015256 |
| Suction UAS | -0.014922 | -0.008952 |
| Reusable scope | -0.028477 | -0.066089 |
| Fibreoptic scope | 0.002355 | -0.040082 |
| Moses Fibre | 0.014154 | -0.031467 |
| TFL | -0.023731 | 0.044175 |
| PCS bleed | 0.060196 | 0.067505 |
| PCS injury | 0.003802 | 0.050603 |
| Postoperative drainage | 0.079495 | 0.011296 |
| Ureteric injury | 0.026067 | 0.032145 |
| Postoperative fever | 1.000000 | 0.208936 |
| Postoperative sepsis | 0.208936 | 1.000000 |
| Residual fragments | 0.059260 | 0.005860 |
| Reintervention | 0.016352 | 0.005675 |
| Day surgery | -0.057900 | 0.007434 |

Table 5. Correlation analysis with infectious complications: Postoperative Fever and Sepsis

1. **STATISTICAL ANALYSIS – LOGISTIC REGRESSION ANALYSIS**


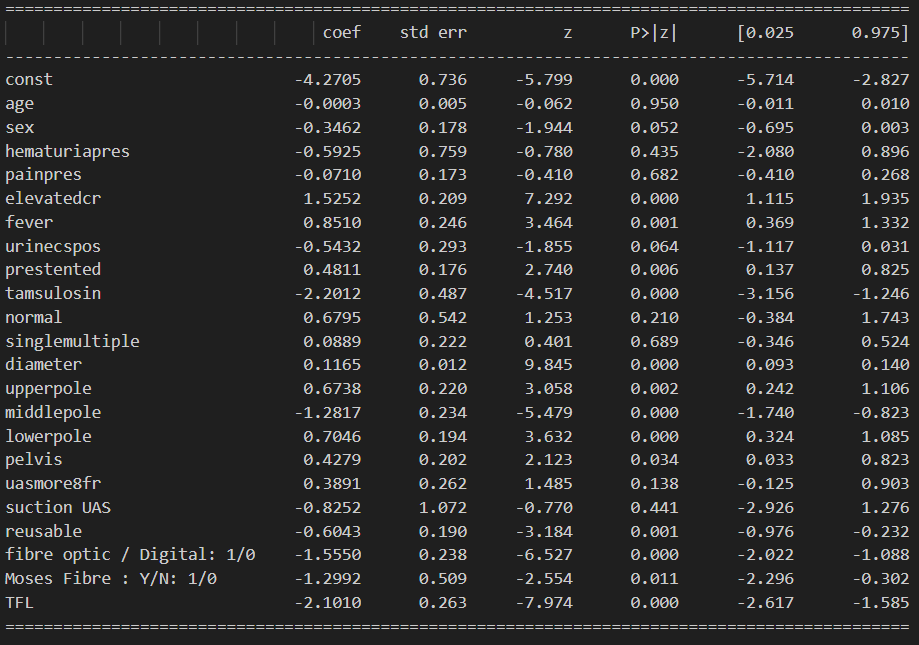


Figure 1. Logistic regression analysis for PCS bleeding


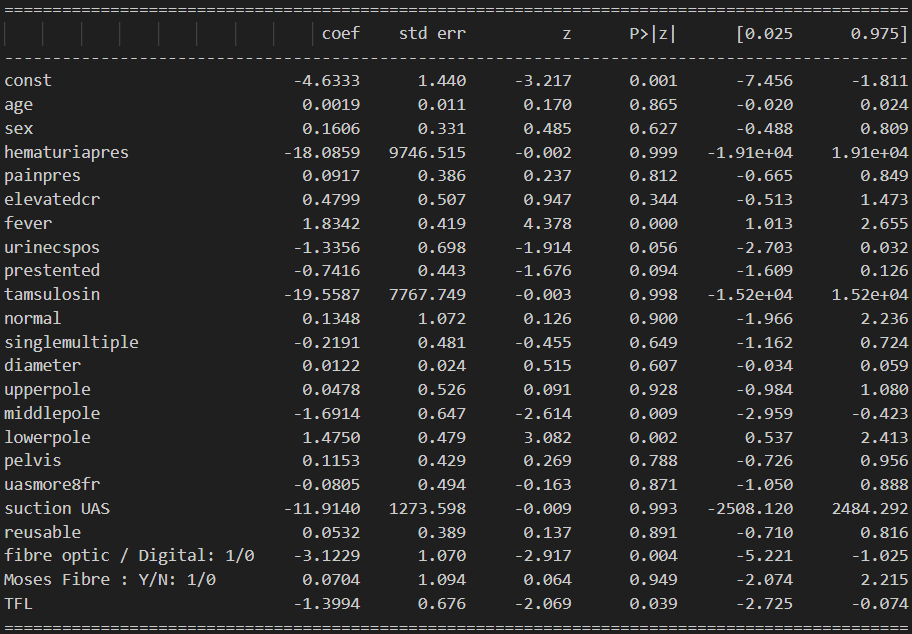


Figure 2. Logistic regression analysis for PCS injury


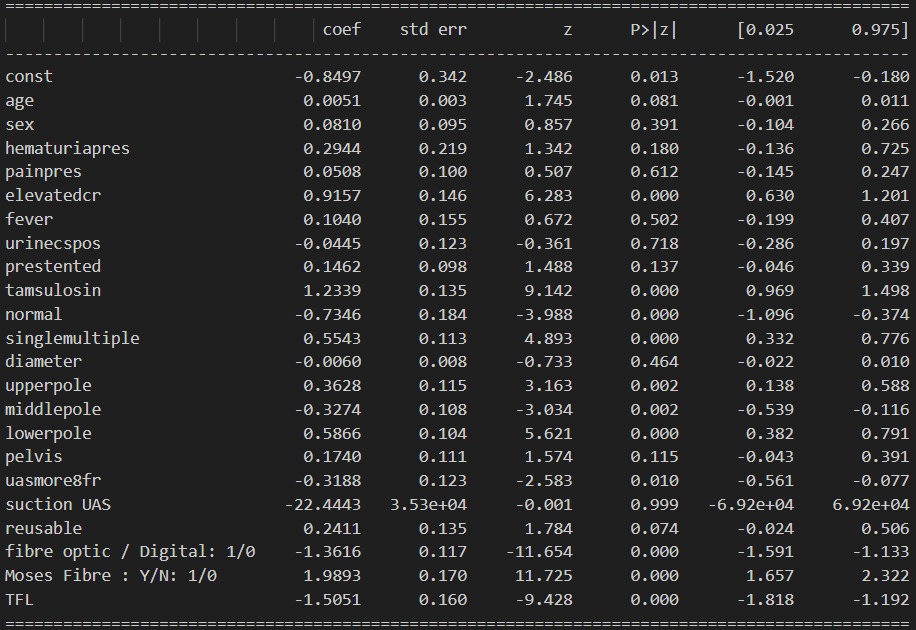


Figure 3. Logistic regression analysis for Postoperative Drainage


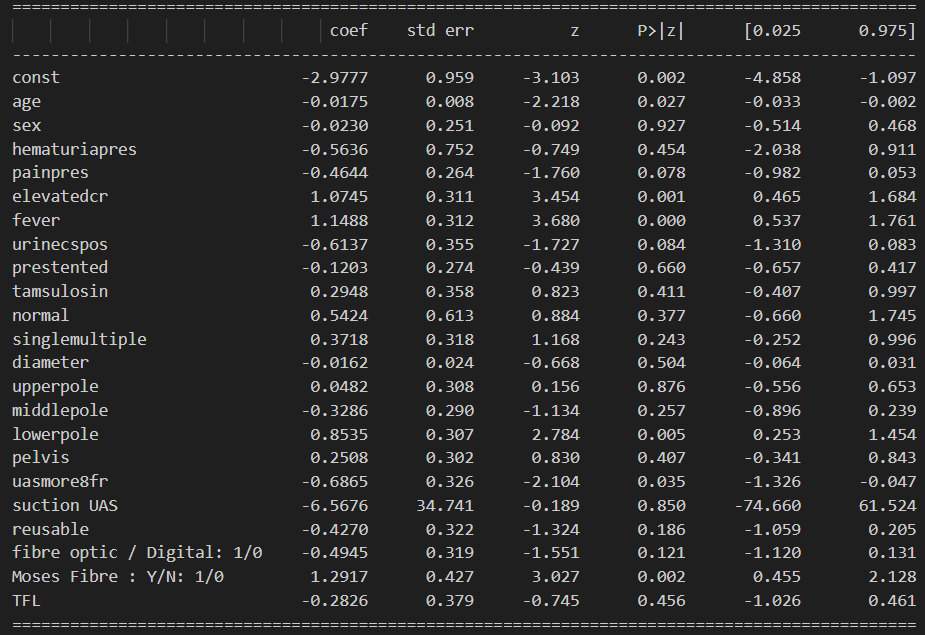


Figure 4. Logistic regression analysis for Ureteric Injury


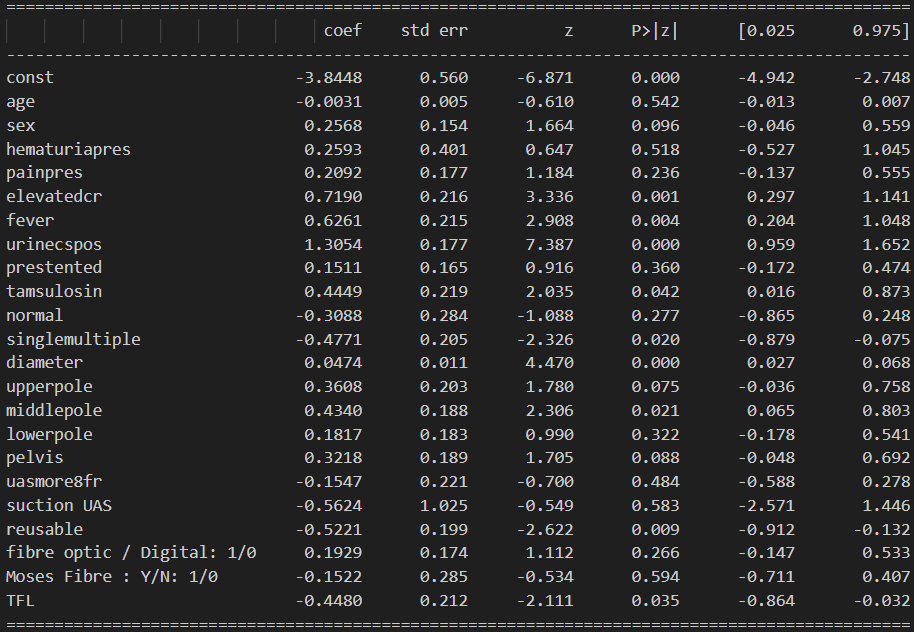


Figure 5. Logistic regression analysis for Postoperative Fever


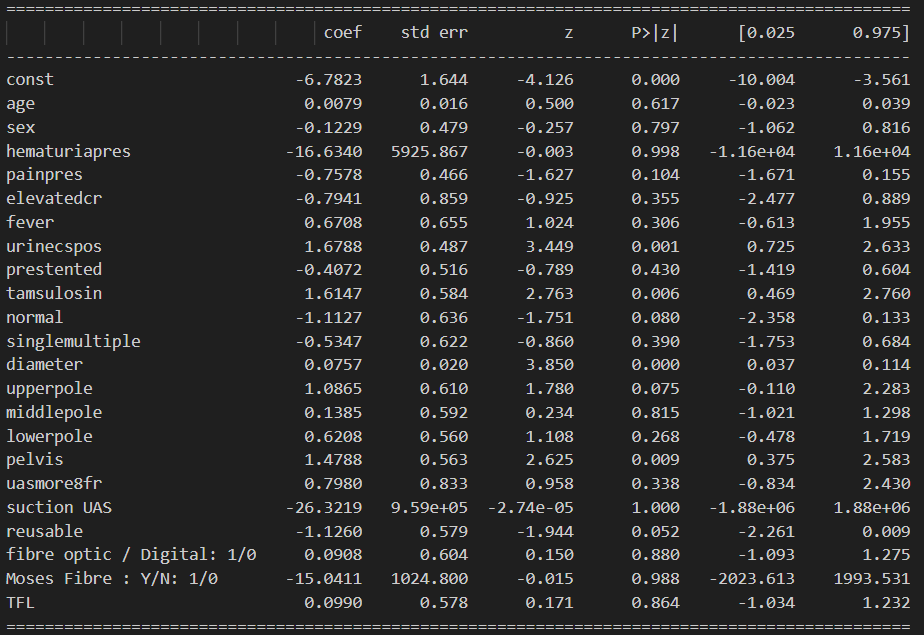


Figure 6. Logistic regression analysis for Postoperative Sepsis

1. **EXPLAINABLE AI**


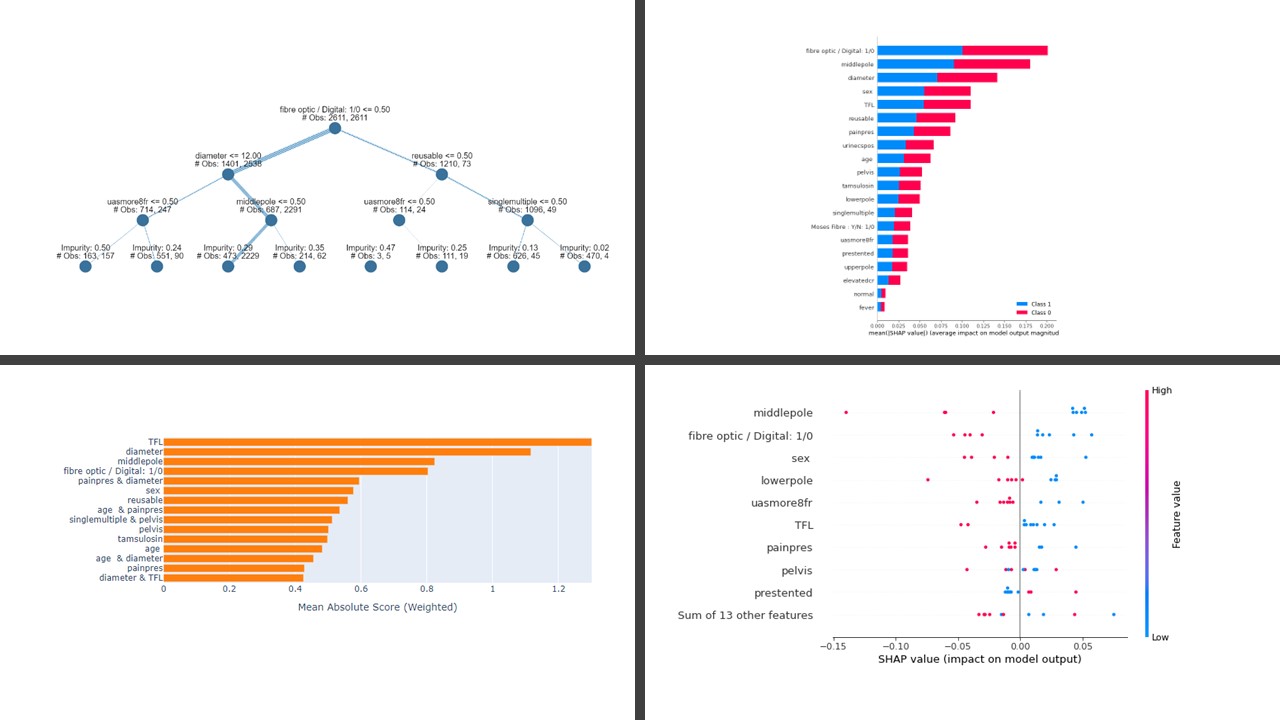


Figure 7. Explainable AI for PCS bleeding. Clockwise from the upper left figure: Explainable Tree, Feature Importance bar chart, Shap summary plot, Shap beeswarm chart


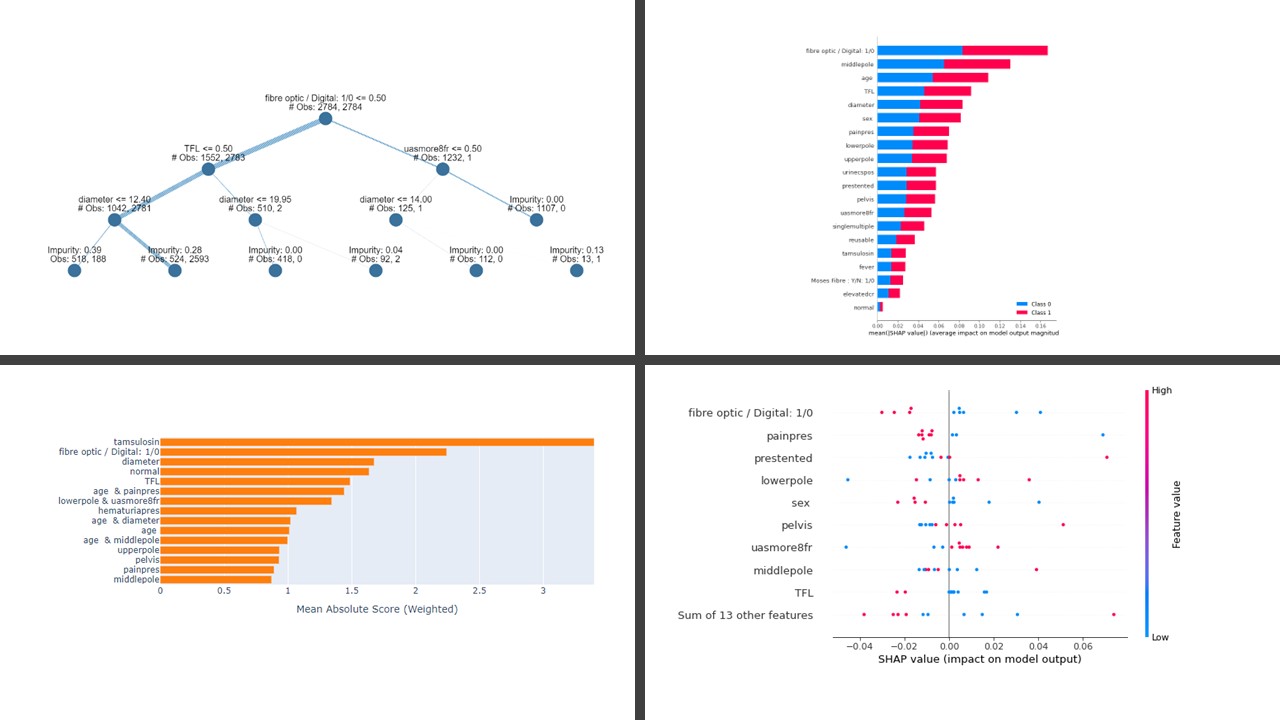


Figure 8. Explainable AI for PCS injury. Clockwise from the upper left figure: Explainable Tree, Feature Importance bar chart, Shap summary plot, Shap beeswarm chart


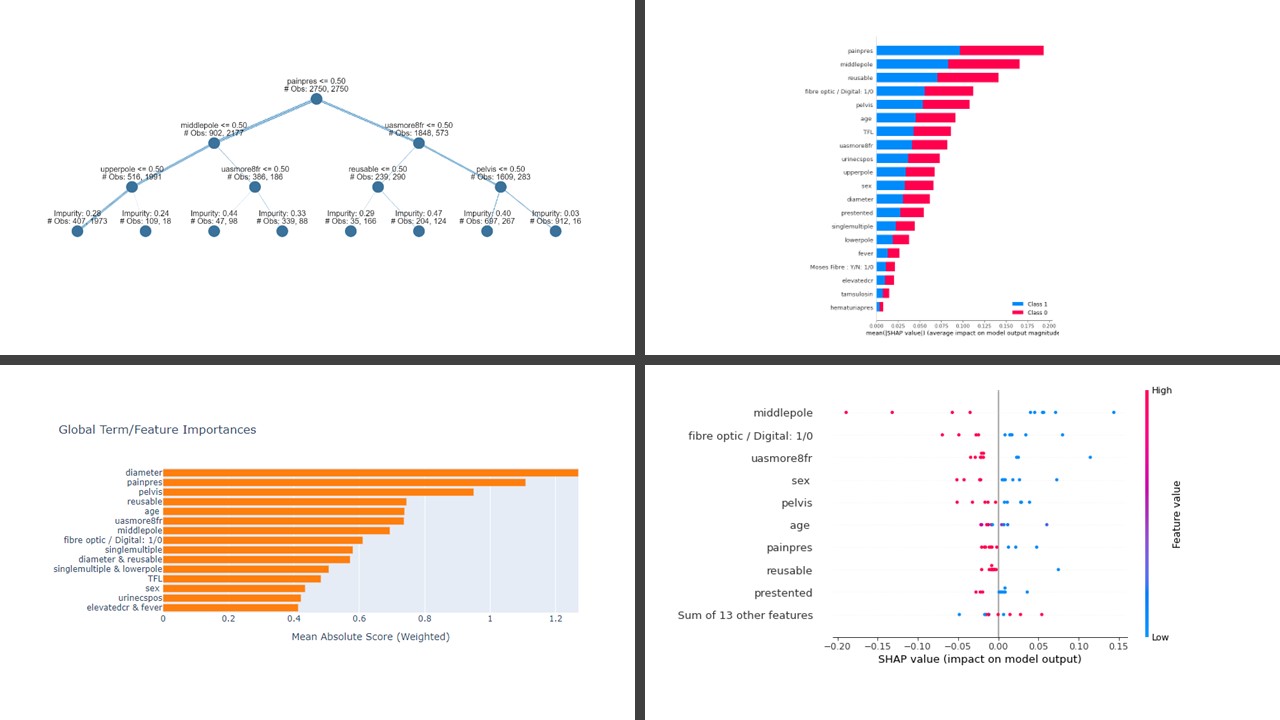


Figure 9. Explainable AI for ureteric injury. Clockwise from the upper left figure: Explainable Tree, Feature Importance bar chart, Shap summary plot, Shap beeswarm chart

1. **MACHINE LEARNING RESULTS**

| **model** | **training-accuracy** | **training-precision** | **training-recall** | **training-f1** | **validation-accuracy** | **validation-precision** | **validation-recall** | **validation-f1** |
| --- | --- | --- | --- | --- | --- | --- | --- | --- |
| naive bayes | 83.51206 | 85.33469 | 83.51206 | 83.29667 | 71.59091 | 58.05746 | 76.55166 | 55.89194 |
| svm-rbf | 83.20567 | 83.7348 | 83.20567 | 83.13955 | 76.70455 | 60.01194 | 80.21199 | 60.11883 |
| knn | 94.88702 | 95.26629 | 94.88702 | 94.87628 | 80.96591 | 59.986 | 75.2785 | 61.59208 |
| svm-poly | 86.53773 | 86.7966 | 86.53773 | 86.51401 | 81.53409 | 61.07409 | 78.29625 | 63.03895 |
| LDA | 89.04634 | 89.67241 | 89.04634 | 89.00296 | 81.67614 | 61.15584 | 78.37282 | 63.17499 |
| QDA | 89.08464 | 89.49455 | 89.08464 | 89.05625 | 82.52841 | 62.16152 | 80.63988 | 64.6007 |
| logistic regression | 90.38682 | 90.57117 | 90.38682 | 90.37589 | 84.09091 | 61.43233 | 75.15539 | 63.98648 |
| svm-linear | 90.71237 | 90.97999 | 90.71237 | 90.69718 | 84.375 | 62.93172 | 79.82764 | 65.92929 |
| adaboost | 91.24856 | 91.44335 | 91.24856 | 91.23827 | 84.51705 | 61.75473 | 75.3851 | 64.43703 |
| Gradient boost | 94.08273 | 94.10375 | 94.08273 | 94.08202 | 88.49432 | 65.9443 | 78.43287 | 69.60195 |
| decision tree | 99.8851 | 99.88522 | 99.8851 | 99.8851 | 91.47727 | 71.01837 | 81.84848 | 74.92163 |
| cat boost classifier | 98.96591 | 98.96824 | 98.96591 | 98.9659 | 94.31818 | 78.34179 | 83.37988 | 80.59965 |
| random forest | 99.8851 | 99.8851 | 99.8851 | 99.8851 | 94.46023 | 79.17094 | 80.74498 | 79.93202 |
| bagging classifier | 99.63615 | 99.63616 | 99.63615 | 99.63615 | 94.46023 | 78.93402 | 82.55262 | 80.60894 |
| Xg-boost | 99.59786 | 99.59949 | 99.59786 | 99.59785 | 94.60227 | 79.23922 | 84.43684 | 81.56966 |
| extra trees classifier | 99.8851 | 99.88522 | 99.8851 | 99.8851 | 95.02841 | 80.98545 | 83.76272 | 82.29974 |

Table 6. ML results for PCS bleeding

| **model** | **training-accuracy** | **training-precision** | **training-recall** | **training-f1** | **validation-accuracy** | **validation-precision** | **validation-recall** | **validation-f1** |
| --- | --- | --- | --- | --- | --- | --- | --- | --- |
| svm-poly | 79.57974 | 84.09366 | 79.57974 | 78.8807 | 60.9375 | 51.69852 | 72.55371 | 41.32747 |
| naive bayes | 89.99641 | 91.14424 | 89.99641 | 89.92615 | 78.40909 | 52.12527 | 70.13247 | 48.63287 |
| svm-rbf | 85.95546 | 86.60196 | 85.95546 | 85.89317 | 79.26136 | 51.84235 | 66.79283 | 48.52564 |
| LDA | 90.8046 | 91.75491 | 90.8046 | 90.75198 | 81.39205 | 53.35817 | 79.1996 | 51.4117 |
| QDA | 92.85201 | 93.11638 | 92.85201 | 92.84104 | 87.35795 | 52.84091 | 67.14349 | 52.53585 |
| svm-linear | 93.26509 | 93.57557 | 93.26509 | 93.25307 | 87.92614 | 53.00691 | 67.43293 | 52.94377 |
| knn | 96.01293 | 96.26132 | 96.01293 | 96.00757 | 88.63636 | 53.83945 | 71.56852 | 54.40267 |
| adaboost | 94.39655 | 94.51235 | 94.39655 | 94.3929 | 90.625 | 54.75292 | 72.58154 | 56.26506 |
| gradient boost | 97.84483 | 97.91694 | 97.84483 | 97.84402 | 93.32386 | 54.08215 | 62.63498 | 55.53585 |
| decision tree | 99.92816 | 99.92819 | 99.92816 | 99.92816 | 95.73864 | 51.75567 | 52.54369 | 52.03488 |
| cat boost classifier | 99.7306 | 99.73205 | 99.7306 | 99.7306 | 96.59091 | 58.09573 | 60.52544 | 59.12917 |
| xgboost | 99.89224 | 99.89247 | 99.89224 | 99.89224 | 96.73295 | 58.64826 | 60.5978 | 59.51089 |
| bagging classifier | 99.85632 | 99.85642 | 99.85632 | 99.85632 | 96.73295 | 53.29961 | 53.05021 | 53.16847 |
| extra trees classifier | 99.92816 | 99.92819 | 99.92816 | 99.92816 | 97.01705 | 54.13545 | 53.19492 | 53.5897 |
| random forest | 99.92816 | 99.92826 | 99.92816 | 99.92816 | 97.72727 | 63.49662 | 57.33051 | 59.42363 |
| QDA | 53.75359 | 74.20014 | 53.75359 | 41.36944 | 97.72727 | 49.07275 | 49.78292 | 49.42529 |

Table 7. ML results for PCS injury

| **model** | **training-accuracy** | **training-precision** | **training-recall** | **training-f1** | **validation-accuracy** | **validation-precision** | **validation-recall** | **validation-f1** |
| --- | --- | --- | --- | --- | --- | --- | --- | --- |
| naive bayes | 73.07273 | 76.8632 | 73.07273 | 72.08812 | 51.42045 | 50.74064 | 58.8776 | 36.41444 |
| svm-poly | 77.34545 | 80.38674 | 77.34545 | 76.76406 | 60.36932 | 50.88484 | 60.18868 | 40.47405 |
| svm-rbf | 82.47273 | 82.74772 | 82.47273 | 82.43585 | 75.56818 | 50.93403 | 58.17126 | 46.23473 |
| LDA | 87.45455 | 87.64188 | 87.45455 | 87.43892 | 79.97159 | 52.19413 | 66.94243 | 49.46005 |
| svm-linear | 88 | 88.11645 | 88 | 87.99083 | 81.39205 | 52.41841 | 67.66812 | 50.24789 |
| logistic regression | 87.32727 | 87.37971 | 87.32727 | 87.32283 | 82.10227 | 52.10915 | 64.7702 | 50.03155 |
| knn | 94.36364 | 94.80847 | 94.36364 | 94.34961 | 82.24432 | 51.68582 | 61.582 | 49.46217 |
| adaboost | 88.76364 | 88.79813 | 88.76364 | 88.76114 | 84.23295 | 52.02274 | 62.59797 | 50.55898 |
| gradient boost | 95.38182 | 95.47131 | 95.38182 | 95.37954 | 89.48864 | 54.16061 | 68.54378 | 55.15152 |
| QDA | 59.92727 | 68.8139 | 59.92727 | 54.56164 | 92.89773 | 52.75356 | 57.24238 | 53.50803 |
| decision tree | 99.89091 | 99.89094 | 99.89091 | 99.89091 | 94.31818 | 56.39547 | 64.4896 | 58.52725 |
| extra trees classifier | 99.89091 | 99.89094 | 99.89091 | 99.89091 | 94.88636 | 51.14601 | 51.73682 | 51.31771 |
| bagging classifier | 99.63636 | 99.63639 | 99.63636 | 99.63636 | 95.45455 | 55.64068 | 58.54862 | 56.72685 |
| random forest | 99.89091 | 99.89101 | 99.89091 | 99.89091 | 96.16477 | 57.4587 | 58.91147 | 58.10909 |
| cat boost classifier | 99.4 | 99.40189 | 99.4 | 99.39999 | 96.30682 | 57.95017 | 58.98403 | 58.43023 |
| xgboost | 99.76364 | 99.76417 | 99.76364 | 99.76364 | 96.875 | 60.67015 | 59.27431 | 59.91718 |

Table 8. ML results for Ureteric Injury

| **model** | **training-accuracy** | **training-precision** | **training-recall** | **training-f1** | **validation-accuracy** | **validation-precision** | **validation-recall** | **validation-f1** |
| --- | --- | --- | --- | --- | --- | --- | --- | --- |
| naive bayes | 69.29758 | 74.41165 | 69.29758 | 67.60073 | 42.75568 | 50.08379 | 50.38944 | 34.11102 |
| QDA | 76.47281 | 79.86127 | 76.47281 | 75.7859 | 56.39205 | 51.86448 | 58.81434 | 42.18317 |
| svm-rbf | 71.3935 | 71.39485 | 71.3935 | 71.39305 | 66.19318 | 51.03191 | 54.34741 | 45.46898 |
| LDA | 79.49396 | 79.5021 | 79.49396 | 79.49254 | 69.74432 | 49.41406 | 47.77906 | 44.84768 |
| logistic regression | 79.43731 | 79.43735 | 79.43731 | 79.4373 | 71.30682 | 49.61812 | 48.60613 | 45.58923 |
| svm-linear | 78.02115 | 78.12904 | 78.02115 | 78.00005 | 72.86932 | 49.13446 | 47.01947 | 45.47615 |
| adaboost | 80.91012 | 80.91383 | 80.91012 | 80.90955 | 73.29545 | 49.90131 | 49.65876 | 46.54863 |
| knn | 92.56042 | 93.35283 | 92.56042 | 92.52627 | 75.85227 | 51.76353 | 55.8396 | 49.61945 |
| svm-poly | 69.93958 | 72.74513 | 69.93958 | 68.98311 | 81.10795 | 51.48407 | 53.7941 | 50.66989 |
| gradient boost | 89.35045 | 89.35943 | 89.35045 | 89.34985 | 82.10227 | 52.24409 | 55.52728 | 51.85724 |
| decision tree | 99.98112 | 99.98112 | 99.98112 | 99.98112 | 87.35795 | 57.45192 | 64.34355 | 59.15275 |
| cat boost classifier | 97.90408 | 97.90769 | 97.90408 | 97.90404 | 89.63068 | 54.85264 | 55.89165 | 55.28292 |
| bagging classifier | 99.58459 | 99.58459 | 99.58459 | 99.58459 | 90.05682 | 56.15143 | 57.32408 | 56.65482 |
| xgboost | 99.60347 | 99.60354 | 99.60347 | 99.60347 | 91.05114 | 57.66566 | 57.8504 | 57.75638 |
| random forest | 99.98112 | 99.98112 | 99.98112 | 99.98112 | 91.33523 | 54.07023 | 53.17332 | 53.51928 |
| extra trees classifier | 99.98112 | 99.98112 | 99.98112 | 99.98112 | 91.33523 | 58.19899 | 58.00077 | 58.0981 |

Table 9. ML results for Postoperative Fever

| **model** | **training-accuracy** | **training-precision** | **training-recall** | **training-f1** | **validation-accuracy** | **validation-precision** | **validation-recall** | **validation-f1** |
| --- | --- | --- | --- | --- | --- | --- | --- | --- |
| QDA | 50 | 25 | 50 | 33.33333 | 0.710227 | 0.355114 | 50 | 0.705219 |
| naive bayes | 80.65093 | 84.37237 | 80.65093 | 80.11264 | 60.9375 | 49.72086 | 40.61516 | 38.20363 |
| svm-poly | 84.1917 | 84.34699 | 84.1917 | 84.17381 | 87.78409 | 50.93414 | 64.06295 | 48.9597 |
| svm-rbf | 85.15737 | 85.19547 | 85.15737 | 85.15335 | 83.52273 | 51.11169 | 71.84549 | 47.94891 |
| LDA | 90.50429 | 90.58283 | 90.50429 | 90.4997 | 85.51136 | 50.17447 | 52.98999 | 47.0505 |
| logistic regression | 92.50715 | 92.55628 | 92.50715 | 92.50499 | 88.21023 | 50.30449 | 54.34907 | 48.03966 |
| svm-linear | 93.0794 | 93.26069 | 93.0794 | 93.07214 | 87.64205 | 50.27266 | 54.06295 | 47.82564 |
| adaboost | 96.67382 | 96.71598 | 96.67382 | 96.67307 | 93.46591 | 50.86022 | 56.99571 | 50.39216 |
| knn | 97.7289 | 97.82448 | 97.7289 | 97.72776 | 92.89773 | 51.81181 | 66.63805 | 51.85732 |
| gradient boost | 99.30258 | 99.30864 | 99.30258 | 99.30255 | 97.86932 | 56.92547 | 69.14163 | 59.98636 |
| bagging classifier | 99.96423 | 99.96426 | 99.96423 | 99.96423 | 98.72159 | 58.0468 | 59.64235 | 58.76879 |
| cat boost classifier | 99.98212 | 99.98212 | 99.98212 | 99.98212 | 99.00568 | 66.45177 | 69.71388 | 67.93128 |
| decision tree | 100 | 100 | 100 | 100 | 98.29545 | 58.87446 | 69.35622 | 62.06897 |
| xgboost | 100 | 100 | 100 | 100 | 98.72159 | 62.28448 | 69.57082 | 65.06203 |
| random forest | 100 | 100 | 100 | 100 | 99.14773 | 66.38136 | 59.85694 | 62.28571 |
| extra trees classifier | 100 | 100 | 100 | 100 | 99.14773 | 66.38136 | 59.85694 | 62.28571 |

Table 10. ML results for Postoperative Sepsis
